# Explora: Interactive Querying of Multidimensional Data in the Context of Smart Cities

**DOI:** 10.3390/s20092737

**Published:** 2020-05-11

**Authors:** Leandro Ordonez-Ante, Gregory Van Seghbroeck, Tim Wauters, Bruno Volckaert, Filip De Turck

**Affiliations:** Department of Information Technology, Ghent University-imec, IDLab, Technologiepark Zwijnaarde 126, 9052 Ghent, Belgium; Gregory.VanSeghbroeck@UGent.be (G.V.S.); Tim.Wauters@UGent.be (T.W.); Bruno.Volckaert@UGent.be (B.V.); Filip.DeTurck@UGent.be (F.D.T.)

**Keywords:** interactive querying, spatiotemporal data, smart city data, sensor data, synopsis data structures, continuous views, microservices

## Abstract

Citizen engagement is one of the key factors for smart city initiatives to remain sustainable over time. This in turn entails providing citizens and other relevant stakeholders with the latest data and tools that enable them to derive insights that add value to their day-to-day life. The massive volume of data being constantly produced in these smart city environments makes satisfying this requirement particularly challenging. This paper introduces Explora, a generic framework for serving interactive low-latency requests, typical of visual exploratory applications on spatiotemporal data, which leverages the stream processing for deriving—on *ingestion time*—synopsis data structures that concisely capture the spatial and temporal trends and dynamics of the sensed variables and serve as compacted data sets to provide fast (approximate) answers to visual queries on smart city data. The experimental evaluation conducted on proof-of-concept implementations of Explora, based on traditional database and distributed data processing setups, accounts for a decrease of up to 2 orders of magnitude in query latency compared to queries running on the base raw data at the expense of less than 10% query accuracy and 30% data footprint. The implementation of the framework on real smart city data along with the obtained experimental results prove the feasibility of the proposed approach.

## 1. Introduction

The increasing pervasiveness of data in the world is currently leading to a new era of human progress, which has been referred to as the *Fourth Industrial Revolution*. As part of this new dynamic, initiatives in the context of smart cities have emerged, aiming at harnessing the power of data to connect with citizens, to build public awareness, to drive urban development and local public policy, and to answer pressing problems such as how to lighten the huge strain that human development has historically placed on the environment and Earth’s natural resources. The burgeoning information technology (*IT*) industry has played a major role in bringing forth these kind of initiatives: big data, Internet of Things (*IoT*), and cloud computing technologies are at the core of the smart city strategies being implemented nowadays around the world [1].

Harrison et al. [2] argue how, by building on the advances in IT, the traditional physical city infrastructure is extended to an integrated framework allowing cities to gather, process, analyze, and make decisions based on detailed operational data. These authors define smart cities through three IT aspects:

- *lInstrumented systems* that enable capturing live real-world data describing the operation of both physical and virtual systems of the city (sensors, smartphones, cameras, and social media, among others.)
- *lInterconnected systems* enabling the instrumented systems to communicate and interact not only among themselves but also with the multiple IT systems supporting the operation of the city’s services.
- *Intelligent systems* able to analyze, model, and visualize the above interconnected data and to derive from the valuable insights that drive decisions and actions to optimize the operation of the city’s services and infrastructure.

Aligned with these aspects, many cities around the world have committed a large amount of resources involving both public and private investment in an effort towards the realization of the smart city vision, yet only few of these initiatives have attained a level of maturity to remain sustainable over time. Research states that one of the key requirements and major challenges for ensuring the sustainability of smart city projects lies in achieving citizen engagement, that is getting communities involved as *prosumers* of the city’s data and services [3,4,5]. This in turn involves providing citizens and other relevant stakeholders with prompt and reliable access to smart city data, enabling them to contribute to the construction and further development of the abovementioned city’s *intelligent systems*.

In this context, data management systems are required to handle the massive amounts of data being continuously generated by smart devices. Typically, said data is defined by spatiotemporal dimensions, e.g., weather, air quality, traffic congestion, parking availability, social media streams, etc. Coping with this large volume of spatiotemporal information while supporting time critical end-user applications—such as those enabling responsive data exploration and visualization—is essentially a big data problem that exceeds the ability of traditional offline data processing methods [6,7]. The nature of this data and requirements of the stated problem call for a more proactive approach where data is *processed during ingestion* in response to recurrent user requests, instead of waiting for it to be accumulated and persisted into an ever-growing database to make it queryable [8,9].

In that sense, the work reported in this paper aims at answering the research question on *how to serve common data exploration tasks over live smart city data coming from nonstationary sensors under interactive (low-latency) time constraints*. To address the stated aim, the approach presented herein explores the use of the stream processing over the sequence of readings coming from mobile sensor devices deployed in an urban environment to aggregate the data of those readings into rich summaries for further querying and analysis. The motivation behind this is two-fold:

1. Typical visual exploration applications for this kind of georeferenced time series present the user with a sort of dashboard containing a map and a number of controls allowing them to perform visual queries on said data on a per region (e.g., by interacting with the map) and a per time period (e.g., by setting an interval of dates) basis [10]. However, these applications are not able to deliver sensible and predictable response times when operating over highly dynamic data such as the raw readings coming from smart city sensors due to its unbounded size: queries can take from several seconds to minutes over a few million sensor measurements. Considering that these queries define restrictions on the spatial and temporal dimensions of data, it is appealing to establish a fragmentation strategy over these two dimensions in order to reduce the cardinality of the search space by computing continuous data summaries. These summaries amount to a fraction of the number of raw observations, allowing data exploration applications to remain responsive to user queries at the expense of some accuracy.
2. These summaries being proactively derived out of the incoming stream of sensor readings enables data management systems to provide client applications with information about the current state of the measured variables without incurring expensive scan operations over the whole raw data. For said summaries to be relevant, frequent user requests as well as interaction patterns when visually exploring spatiotemporal data should be considered to drive the design of the stream processing pipeline and to determine which technologies could support its operation. By abstracting a generic framework embracing these requirements, it is possible to test to what extent existing data technologies support time-sensitive applications and to estimate their limitations in terms of scalability and reliability.

Aligned with these considerations, the main contributions of the work introduced in this paper are (*1*) the formulation of a technology-agnostic approach for the continuous computation of data summaries over a live feed of sensor readings by applying a spatiotemporal fragmentation scheme to the sequence of observations, (*2*) the formal definition of a uniform interface for querying said summaries based on recurrent user interaction patterns, and (*3*) the realization of the proposed approach by implementing a complete stream processing pipeline able to operate over real-world sensor readings coming from a smart city setup deployed in the city of Antwerp in Belgium. For this, a number of existing open source data technologies running on commodity hardware have been used, being able to test their ability to serve visual exploration applications under different configurations. Results show that, by implementing the proposed continuous aggregation approach on centralized and distributed data stores, it is possible to outperform a traditional time series database bringing down query response times by up to two orders of magnitudel, reaching sub-second performance for requests made over one year’s worth of data (nearly 14+ million observations). This document provides a detailed description of the components and design decisions behind the definition of this approach. Section 2 addresses the related work. Section 3 focuses on the main contribution of this work and elaborates on the framework for supporting data exploration on spatiotemporal data through continuous computation of data summaries. Section 4 describes the implementation of the proposed approach, while Section 5 discusses the experimental setup and results. Finally, conclusions and pointers towards future work are provided in Section 6.

## 2. Related Work

Recent surveys on big spatiotemporal data by Yang et al. [11] and He et al. [12] argue that most of the existing tools for visual exploration serve a single specific use case, acknowledging the need for more flexible data visualization approaches that allow users to examine the behavioral changes in the information over the temporal and spatial domains while having sensible storage requirements and improving query performance. The approach described in this paper has been precisely formulated to comply with those requirements, considering smart cities as a meaningful use-case scenario. This section discusses existing literature regarding spatiotemporal data management, visual exploratory analysis on smart city data, and big data frameworks for smart cities.

### 2.1. Spatiotemporal Data Management

The problem of speeding up spatial queries has been studied extensively from the data management perspective. Ganti et al. [13] propose *MP-trie*, a mechanism that reduces the problem of spatial indexing to that of prefix-matching over binary strings by encoding spatiotemporal data into a data structure they call *Space-Time Box* (STB) [14]. According to the authors, *MP-trie* provides a 1000× performance improvement over traditional indexing approaches (specifically R*-tree [15]), though it only reaches said performance when implemented using hardware acceleration (*ternary content-addressable memory* or *TCAM* [16]). *MP-trie* is described as an indexing mechanism intended to speed up spatial queries such as finding all the objects within a distance *r* from a point *p* (*range queries*) or finding the *top-K* nearest neighbors from *p* (*kNN queries*). Similarly, *SATO* [17] and *AQWA* [18] proposed by Vo et al. and Aly et al., respectively, are two data-synopsis-based mechanisms aiming at finding the optimal partitioning scheme in order to lower the response time of spatial queries in distributed spatial datasets. However, these and other similar approaches dealing with spatial indexing and partitioning [19,20,21] overlook the temporal dimension of the data typical of smart city applications and, in consequence, might fall short in supporting requests intended to explore the historical behaviour from a given sequence of observations.

This issue has also been addressed in the context of *Wireless Sensor Networks* (WSN). Wan et al. [22] present a promising technique for high-dimensional indexing of the sensor data produced within large WSNs, based on the *Voronoi Diagram* data structure. The mechanism that Wan et al. propose includes a hierarchical *in-network* storage which is capable of answering different range monitoring queries, based on the devised indexing scheme. However, given the restrictions in terms of power, storage, and computing resources typical of WSN nodes, pushing a large volume of queries down to the sensing devices for processing could compromise the availability of the network. The approach proposed in this paper deals with delivering interactive-level performance for basic exploratory tasks. In this use case, it is not uncommon to serve multiple users, each one issuing several queries during a session of data exploration, which would entail a prohibitive computational expense for a WSN.

### 2.2. Visual Exploratory Analysis on Smart City Data

Research on visualization techniques for interactive exploration of smart city data is mainly focused on enhancing user experience by providing them with responsive client-side applications. Doraiswamy et al. [10] proposed *Raster join*, a technique to speed up spatial join queries supporting the interactive exploration of multiple spatiotemporal data sets at the same time. The *Raster join* technique—that leverages current generation graphics processing units (GPU)—was integrated to *Urbane* [23], a 3D visual framework to support the decision making for designing urban development projects. By integrating the proposed technique, *Urbane* is able to handle requests over hundreds of millions of observations with nearly sub-second performance. Similarly, Murshed et al. [24] introduced a web-based application for analysis and visualization spatiotemporal data in smart city applications called *4D CANVAS*. This application enables users to perform interactive exploration on both space (*3d*) and time dimensions over a data set stored on disk by leveraging on a WebGL-based framework known as *Cesium* [25]. Also, under these visual data exploration approaches, Li et al. developed *SOVAS* [26], a visual analytics system for query processing of massive array-based climate data, which works on top of *Hadoop* and provides an SQL-based language for users to express their information needs and to conduct spatial analytics tasks. One common feature platforms described in this section (and related solutions like References [27,28]) do not incorporate is the ability to process data in a streaming format. These solutions expect the spatiotemporal data they operate on to be residing on the file system (whether local or distributed), some of them requiring additional offline preprocessing to be able to deliver the functionality they advertise.

In contrast to the approaches above, Cao et al. present a visual interactive system known as *Voila* [29], able to process a stream of traffic flow data and to assist users in detecting anomalous events. *Voila* assigns an anomaly score for a given region at a certain point in time by examining changes in patterns’ occurrence-likelihoods. Then, users can indicate whether the system has accurately identified anomalous events, and *Voila* incorporates their judgement, recomputing the anomaly scores by using a bayesian approach. In the same vein, Chen et al. proposed *ADF* [30], an open framework for anomaly detection over fine particulate matter measurements (PM2.5), coming from a network of low-cost sensors rolled out on an urban environment. The *ADF* framework is able to identify spatiotemporal anomalous sensor readings as new data comes in, thanks to a statistical-based method called *time-sliced anomaly detection* (TSAD), which thrives on contrasting the readings from each sensing device with those from neighboring sensors to detect and label atypical observations. While the systems proposed by Cao et al. and Cheng et al. were designed with the anomaly-detection use case in mind, the approach described herein was devised for serving a more general purpose, i.e., enabling basic exploratory analysis tasks on live smart city data—regardless of the kind of environmental information being ingested, the number or type of sensor devices, or their location (fixed or mobile)—considering both spatial and temporal data dimensions under interactive response time constraints. It is worth mentioning that one of the main features of the approach introduced in this paper is that of being an extensible, technology-agnostic data analysis pipeline, and as such, it would be able to integrate the anomaly detection methods implemented in systems like *Voila* and *ADF* while offering interactive querying capabilities over their resulting outcome.

### 2.3. Big Data Frameworks for Smart Cities

As stated earlier, handling spatiotemporal data in the context of smart cities is inherently a big data problem which has become a prolific research field over the last few years. This section addresses some recent advances and initiatives in this regard. Osman A. proposes the *Smart City Data Analytics Panel (SCDAP)* [31], a framework for big data analytics tailored to the specific requirements of smart city environments. *SCDAP* has been laid out in a 3-layered architecture encompassing multiple stages in the data analysis pipeline ranging from data acquisition, cleansing, and transformation to online and batch data processing, including the management and aggregation of data analysis models serving smart city applications. The author outlines a prototype implementation of a big data analytics platform adopting the artifacts defined in *SCDAP*, using a number of existing open source technologies. However, no indication is provided with regards to its actual application and performance on real or synthetic smart city data.

Badii et al. [32,33] introduce *Snap4City*, a visual programming environment along with a suite of microservices allowing users to create event-driven IoT applications in the context of smart cities. The platform runs on top of *Node-RED* [34] and offers a comprehensive set of visual constructs through which users can assemble complex data flows supporting smart city applications (dashboards, route planning, data analytics, etc.). Another platform intended to facilitate the development of smart city applications is *InterSCity* proposed by Del Esposte et al. [35]. *InterSCity* also advocates for a microservice architecture and provides a Web service middleware that enables the integration of heterogeneous IoT devices, services, and resources. While enabling interactive data exploration is not the main concern of platforms like *Snap4City*, *InterSCity*, and other similar approaches [36], their focus on microservices allows for the integration of data management solutions like the one presented in this paper, aiming at supporting time-sensitive smart city applications.

Aguilera et al. [37] propose *IES Cities*, a data integration platform that enables the creation of citizen-centered applications in the context of smart cities. This approach is founded on the premise that the smart city vision should be achieved through the organic coalescence of government data (*linked open data*), IT infrastructure in place throughout the city (*IoT*), and citizen initiative and contributions mediated through smartphone applications (*crowd-sourced data*). While the *IES Cities* platform is able to integrate smart city data sourced in structured formats such as RDF, JSON, and CSV and relational databases, it does not specifically tackle the issue of enabling interactive data exploration over live streams of spatial-time series data being continuously produced within a smart city environment.

## 3. Explora: Interactive Exploration of Spatiotemporal Data through Continuous Aggregation

The previous section discussed existing approaches addressing the issue of handling spatiotemporal data to support visual exploratory applications in the context of smart cities. Most of the studies in this review tackle specific aspects of the problem, neglecting in some cases the time dimension of the data; others deal with mechanisms for optimizing display and interaction features, but fall short when processing data as it comes in; and others are concerned with frameworks and guidelines for building smart city applications from the perspective of big data. The proposal addressed in this paper builds on top of the mentioned approaches and introduces a generic framework called Explora (***E**fficient e**XPLOR**ation through **A**ggregation*) intended for speeding up spatiotemporal queries supporting visual exploratory analysis conducted on mobile sensor data. This section discusses the key requirements and features driving the design of the devised framework, then introduces the enabling techniques adopted to support the framework requirements, elaborates on the framework components and architecture, and finally details the formal definition of the data processing pipeline lying at the core of the framework.

### 3.1. Framework Requirements and Features

User interaction patterns typical in visual data exploration have been identified in a former study by Andrienko et al. [38], distinguishing two main categories of exploratory actions on spatiotemporal data: (*i*) *elementary tasks*, aiming at describing the state of the observed variable(s) at a particular instant (*time*) over a given region (*space*), and (*ii*) *general tasks*, intended for describing how the state of the observed variable(s) in a given region (*space*) changes over *time*. By composing these basic tasks, it is possible to support more elaborate workflows to help answer different questions about the data at hand. This is why the set of categories by Andrienko et al. has become commonplace benchmark tasks to assess the quality of user interactive exploration on spatiotemporal data [39]. On the other hand, a related study by Liu and Heer [40] addressing the effects of latency on visual exploratory analysis states that *high delay reduces the rate at which users make observations, draw generalizations, and generate hypotheses*. Considering these findings, two key requirements have been derived to drive the design of the Explora framework proposed herein:

**R1.**  Support elementary and general visual exploratory tasks on spatiotemporal data generated by mobile sensors in a smart city setup.
**R2.**  *Provide fast answers (sub-second timescales as target) to queries serving the two basic visual exploratory tasks stated in***R1.**

In addition to these key requirements—and following the steps of several of the big data frameworks for smart cities discussed earlier in this document—a microservices approach has been adopted to profit from features such as *modularity*, *extensibility*, and *scalability*. As a generic framework, Explora should be able to incorporate different sources of sensor readings as well as multiple methods for storing, partitioning, and querying said data. Microservices advocate for establishing a clear separation of concerns and for identifying the functional building blocks that support the framework capabilities. This *componentization* facilitates the overall system development and deployment and further promotes other appealing features such as *extensibility* and *maintainability*, reducing the amount of effort required to introduce modifications, since it would involve making said changes to certain individual microservices.

Likewise, an implementation of Explora should be flexible enough to cope with the increasing volumes of sensor data coming in as well as seasonal load variations (e.g., user activity and data influx are expected to peak during certain time periods). The effective modularization into independent deployable components enables these implementations to elastically react to system load; this is, they are able to dynamically *scale-up* or *down* the number of microservice instances they need to efficiently deal with the volume of requests at a given moment.

Lastly, as a consequence of adopting a microservices approach, the Explora framework benefits from two other highly desirable features, namely *availability* and *portability*. By relying on microservices, the framework components are designed to be self-contained and interchangeable, which helps in timely spotting system failures when they occur, introducing changes to the relevant components and redeploying them without incurring in any major system downtime. Microservices also encourage the use of well-defined interfaces exposing the capabilities of each component and mediating the interaction with other system modules and the underlying infrastructure. This way, as long as modules comply to said interfaces, details such as the language they are written in and the software frameworks they use are not relevant.

### 3.2. Enabling Techniques

To comply to the committed requirements, the Explora framework relies on two enabling techniques: query categorization and data synopsis.

#### 3.2.1. Query Categorization

**Figure 1:**
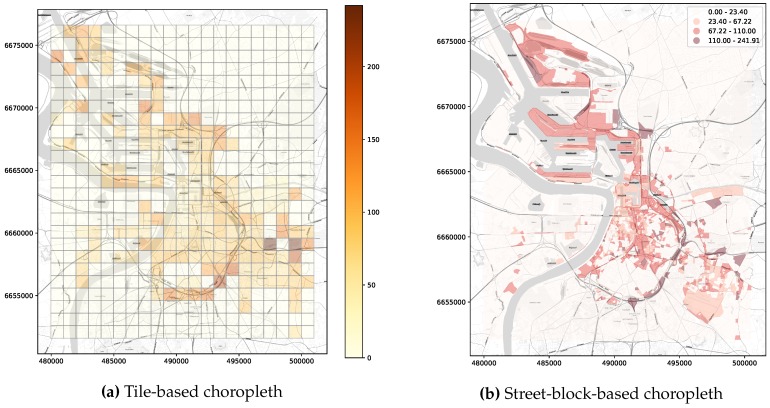
Examples of visualizations expected from an *elementary* exploratory task: These choropleth maps show the concentration of nitrogen dioxide ($NO_{2}$) over the city of Antwerp, BE through a one-month period.

Requests serving *elementary* and *general* exploratory tasks (requirement **R1.**) query spatiotemporal data on different attributes and satisfy different information needs. When conducting *elementary tasks*, users are interested in visualizing the state of the observed variable over a particular region at a given moment in time. For instance, a user might want to know the concentration of particulate matter (PM) over their neighbourhood during peak hours. Queries serving these kind of tasks expect the requested time (in terms of *timestamps*) and the geographic area of observation (in terms of *longitude* and *latitude*) as input parameters and provide as output a sort of snapshot accounting for the value of the observations aggregated over discretized units of space covering the region of interest. Typical examples of the kind of visualizations that might be presented to the user as a result of these *elementary* exploratory tasks are the choropleth maps shown in Figure 1. Queries falling into this category have been labeled as *Snapshot-temporal queries* (ST).

**Figure 2:**
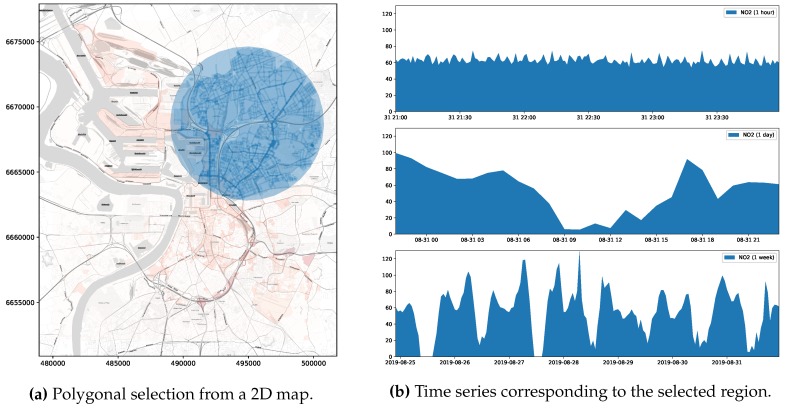
Example of a *general* exploratory task: (**a**) the visual query prompted by the user: *How have the $NO_{2}$ emissions historically evolved within the traced perimeter?* (**b**) Three time series charts reporting on the concentration of the $NO_{2}$ over the past one hour, 24 h, and one week.

On the other hand, the intent behind *general* exploratory tasks consists in comparing the state of the observed variable over a given region along different points in time. Users might conduct these kind of tasks, for instance, by selecting an arbitrary geographic area on a map and by choosing the period of time they are interested in reviewing. This way, queries supporting *general* exploratory tasks expect as inputs a specification of the region of interest along with the inspection time period and yield as answer the value of the observed variable aggregated over discretized units of time (minutes, hours, days, etc.), revealing the historical behaviour of the measured variable. Queries belonging to this category are referred to as *Historical-spatial queries* (HS). Figure 2 below outlines a common outcome of a *general* exploratory task.

**Figure 3:**
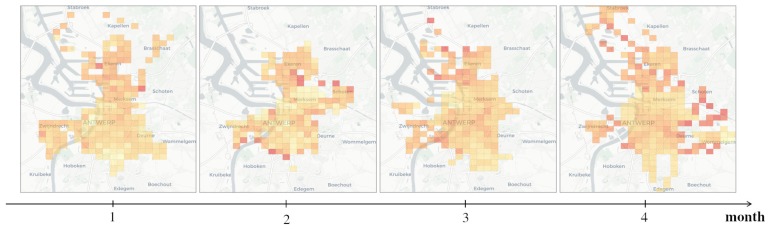
Example of a *general* exploratory task as a composite of multiple *elementary* tasks.

It is worth noting that a *general* exploratory task can be fulfilled as well by sequentially executing multiple *elementary* tasks. Consider for instance the case in Figure 3, where a progression of choropleths is displayed, as the result of running a series of *snapshot-temporal* queries requesting the state of the observed variable over several months. While in practice this sequence of requests serve a *general task* intent, in cases like this, the proposed framework deals with each individual query in isolation, regardless of the overall purpose of the exploratory task.

#### 3.2.2. Data Synopsis and Spatiotemporal Fragmentation

Both *historical-spatial* and *snapshot-temporal* queries are expensive and time-consuming when running on large spatiotemporal data, since they involve executing demanding scan, sort, and aggregate operations. Reducing the data-to-insight time in visual exploratory applications requires speeding up this kind of query (requirement **R2.**). The temporal and spatial dimensions of smart city data along with the specific features of HS and ST queries make this problem appealing for synopsis data structures [41]. Synopsis structures are by definition substantially smaller than the base data set they are derived from. They represent a summarized view of the original data intended to serve certain predefined types of queries. In a streaming setting, synopsis structures are created as data comes in; this way, users can submit queries on the data stream at any point in time and get prompt (and often approximate) answers based only on the data available thus far in the synopsis structures.

**Figure 4:**
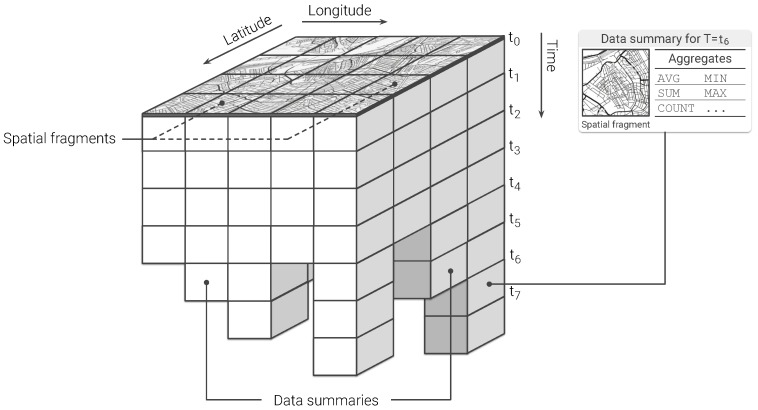
Spatiotemporal fragmentation for continuous computing of data summaries.

As stated in the previous section, the outcome of queries supporting visual exploratory applications is typically delivered in discretized units of time (HS queries) or space (ST queries). Explora takes advantage of such discretization to assemble synopsis structures—namely, *continuous views*—that are incrementally computed as new sensor observations arrive. Consider the choropleth maps presented back in Figure 1, reporting on the concentration of nitrogen dioxide ($NO_{2}$) over the city of Antwerp, Belgium, for a period of one month. To build these visualizations, raw sensor readings occurring during the requested period are aggregated according to the *spatial fragment* (i.e., tile/street-block) which they fall into. Then, the value of said aggregate is encoded in the color displayed for each fragment, providing the user with insight about the state of the observed variable. Similarly, the time series charts shown in Figure 2b are laid out by aggregating raw observations into specific time resolutions or bins (i.e., minutes, hours, and days) in correspondence to the time said observations occurred. Instead of computing these aggregates on request over the raw sensor observations, Explora sets a spatial fragmentation schema upfront and applies multiple aggregate operations (e.g., *average*, *sum*, and *count*) for a number of time resolutions (from one-minute to monthly) over the incoming stream of sensor readings. The collection of aggregates corresponding to an individual spatial fragment over a single time bin has been labeled as *data summary*. This way, continuous views are assembled for each of the supported time resolutions by persisting the resulting data summaries into an structure that can be seen as a sort of *dynamic spatiotemporal raster*. Figure 4 below shows a schematics of this structure, in which a regular tile grid is used as spatial fragmentation strategy for illustrative purposes.

**Figure 5:**
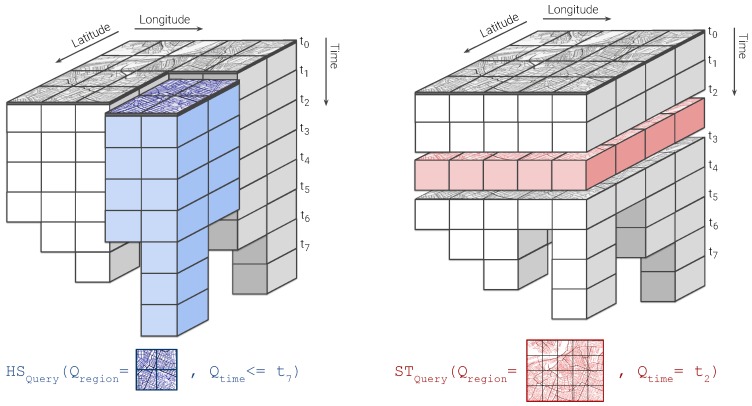
Query resolution on continuous views: The diagram on the left describes an HS query requesting the historical behaviour of the observed variable over $Q_{\mathrm{region}}$, while the one to the right shows an ST query requesting the state of the observed variable at instant $Q_{\mathrm{time}}=t_{2}$.

Notice that, regardless of the volume of sensor readings being ingested, the size of the continuous views only depends on the size of the spatial fragments and time bins being used, this is, the lower the resolution of the spatiotemporal fragmentation scheme, the smaller the size of the corresponding view. By querying these synopsis structures instead of the raw sensor data, users of visual exploratory applications can experience a more responsive feedback at the expense of some accuracy. It is worth noting as well how, thanks to the way these continuous views are structured, answering HS and ST queries comes down to cutting longitudinal (i.e., along the *time* axis) and transverse slices (i.e., along the *longitude/latitude* plane), respectively, and further aggregates their constituent data summaries afterwards, as illustrated below in Figure 5.

### 3.3. The *Explora* Framework: Components and Architecture

**Figure 6:**
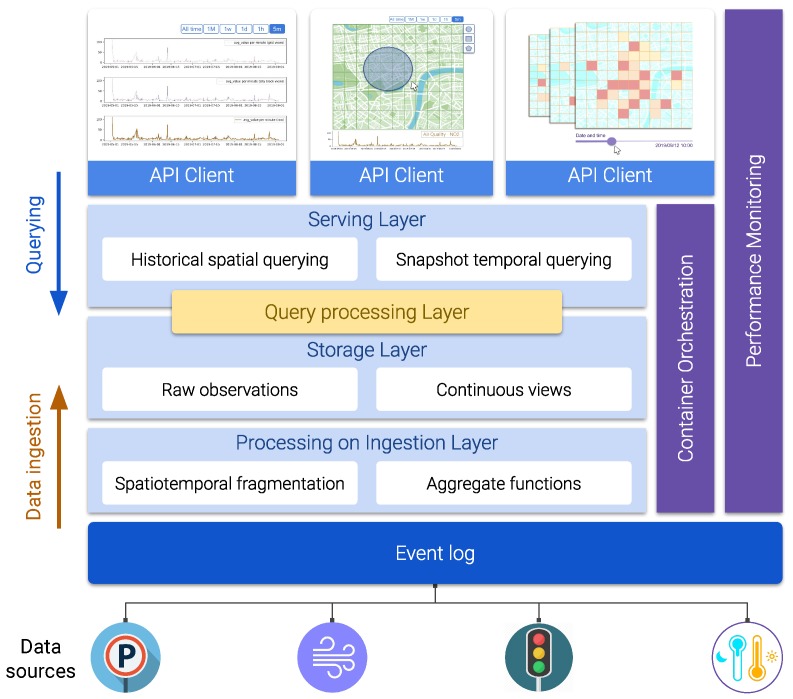
Components and architecture of the Explora framework.

This section deals with the definition of the framework’s building blocks and how they fit together to meet the requirements stated earlier. As Figure 6 illustrates, the Explora framework adopts a layered architecture approach, where functional modules are organized into logical tiers, namely *processing on ingestion*, *storage*, *query processing*, and *serving* layers. Besides these functional layers, three supporting layers are defined for decoupling the system from the available sensor data sources (*event log*) and for providing monitoring capabilities and infrastructure resources for the components in the functional tiers to operate with (*performance monitoring* and *container orchestration*). The description of these layers and their associated components is addressed next.

***Event*** ***log*** This layer serves as an interface between the framework and the sensor data providers. It collects the raw sensor data and hands it over to the upper layers for scalable and reliable consumption and further processing. This tier can be realized through a distributed append-only log that implements a *publish–subscribe* pattern, allowing data producers to post raw sensor observations to logical channels (topics) that are eventually consumed by client applications in an asynchronous way.
***Processing*** ***on*** ***ingestion*** This layer subscribes to the *event log* to consume the stream of raw sensor observations and processes them to continuously generate the data synopsis structures that the framework thrives on. The stream processing mechanism this layer implements is subject to the particular designated *spatiotemporal fragmentation* strategy and the set of supported *aggregate functions* used to compute the corresponding data summaries. This layer represents one of the core components of the Explora framework, as it comprises the modules in charge of applying the ingestion procedure that will be further discussed later in this section (Algorithm 1).
**Storage** **layer** This tier comprises the artifacts responsible for providing persistent storage for both the *continuous views* generated in the ingestion layer and the stream of *raw sensor observations* being consumed from the *event log*, along with the corresponding programming interfaces (APIs) for enabling modules in adjacent tiers to conduct basic data retrieval tasks. Complex requests—such as those supporting the *elementary* and *general* exploratory tasks discussed back in Section 3.1—might be handled in cooperation with the *serving layer* at the top, depending on querying capabilities offered by the data storage technologies implemented in this layer.
**Serving** **layer** This tier provides an entry point for visual exploratory applications to interact with the framework and to access the available sensor data. The serving layer implements a uniform API allowing client applications to issue *historical-spatial* and *snapshot-temporal* queries against the data persisted in the storage layer (both raw observations and continuous views). Depending on the storage technologies used in the underlying *storage layer*, the serving tier might also take part in the query resolution process. This is why *query processing* is represented as a separate layer, sitting in between the two upper tiers.
**Query** **processing** As stated above, responsibilities of this tier overlap those from the contiguous layers (*serving* and *storage*). The processing performed in this layer supports query answering for both *historical-spatial* and *snapshot-temporal* inquiries (according to the procedures detailed in Algorithms 2 and 3, discussed later in Section 3.4). Where this processing takes place is determined by the capabilities of query API provided by the data storage being used. Thus, for instance, a data store offering an expressive SQL interface would be able to handle most of the query processing tasks, while a typical *key-value* store offering simple lookup operations would require a large part of the query processing to be performed programmatically in the *serving layer*.
**Container** **orchestration** All the functional components of the Explora framework are implemented as containerized microservices. The *container orchestration layer* is in charge of the automatic deployment, scaling, load balancing, networking, and life-cycle management of the containers that these components operate on. Examples of existing technologies able to support the functionality required from this layer are *Kubernetes* [42]—deemed as the *de facto* standard for container orchestration to date—*OpenShift* [43], and *Apache Mesos* [44].
**Performance** **monitoring** The role of this layer is to keep track of a number of metrics accounting for the computing requirements (memory and CPU usage) and overall performance of a system implementing the Explora framework (query response time and accuracy). To that end, this layer relies on tools provided by the *container orchestrator*, the operating system, and third-party libraries for statistical analysis and data visualization. Performance information such as that reported later in Section 5 is compiled in this layer.
**Client** **applications** Finally, visual exploratory applications consume the API available through the *serving layer* to support different data exploration use cases based on the two abstracted categories of exploratory tasks: *elementary* and *general*. Section 4 provides a number of examples of said use cases, presented as part of proof-of-concept implementations of the proposed framework.

### 3.4. The *Explora* Framework: Formal Methods and Algorithms

The formal definition of the query resolution mechanism along with the ingestion procedure at the core of the Explora framework are detailed next.

#### 3.4.1. Data Ingestion: Continuous Computation of Data Synopsis Structures

Let us represent a mobile sensor observation (reading) as the following tuple:

(1) $$r=\langle t,x,y,s,v,a_{0},a_{1},\ldots ,a_{n}\rangle$$

where *t* is the *timestamp* indicating when the observation was made, *x* and *y* being respectively the *longitude* and *latitude* where the observation took place; *s* is the *observed (sensed) variable*; *v* is the *observed value* as measured by the sensor; and $a_{i}$ is additional attributes and metadata (device identifier, measurement units, etc.).

Then, a continuous view for a given observed variable, *s*, can be represented as a function V that maps a spatial fragment, ϕ, and a temporal bin, τ, to its corresponding data summary σ (collection of aggregates), as follows:

(2) $$V_{\langle s,\Phi ,\Omega \rangle }:\left(\phi ,\tau \right)\mapsto \sigma ;\;\sigma =\left\{\sigma_{\mathrm{AVG}},\sigma_{\mathrm{SUM}},\sigma_{\mathrm{COUNT}},\ldots \right\}$$

where ϕ is one of the discretized units of space into which a geographic area is partitioned, according to a certain spatial fragmentation strategy Φ (e.g., tiles, hexagons, street blocks, etc.) and τ identifies one of the temporal buckets resulting from setting a regular frequency, Ω, at which the incoming sensor observations are aggregated (e.g., minutely, hourly, daily, etc.).

Similarly, the mechanism for assigning a sensor observation *r* to its corresponding data summary can be defined as a function F that takes the spatial and temporal attributes from *r* and returns the spatial fragment and temporal bin identifying the data summary to which *r* belongs:

(3) F:r〈t,x,y〉↦(ϕ,τ)

With these definitions in place, the formal procedure for data ingestion in Explora is presented below in Algorithm 1. The process starts by first setting a spatial fragmentation strategy ($\Phi_{k}$), a frequency of aggregation ($\Omega_{k}$), and a set of aggregate methods to be supported ($\Sigma_{k}$) (lines 3–6). Then, persistent storage for a new continuous view is allocated (assuming it does not exist yet) (line 7), and the sensor observations coming from a stream S are taken in, one after the other. To determine the data summary into which each sensor reading has to be aggregated, the spatial fragment and temporal bin are computed by applying the function F on each of the incoming readings. With this input, the corresponding data summary is retrieved from the view (lines 9 and 10). Then, the collection of aggregates from the data summary gets updated and the changes are persisted in the continuous view (lines 11–19). In practice, the type of each one of the aggregate functions in $\Sigma_{k}$ (i.e., *distributive*, *algebraic*, or *holistic* [45]) determines how the update procedure in line 16 is implemented. Later, in Section 4, two prototypes are presented for illustration and proof of concept.
**Algorithm 1** Explora ingestion procedure.1: Let S be a stream of sensor observations of a variable $\hat{s}$2: $S=\left\{r_{0},r_{1},r_{2},\ldots \right\};\;r_{i}=\langle t_{i},x_{i},y_{i},\hat{s},v_{i},a_{0i},a_{1i},a_{2i},\ldots \rangle$▹ Unbounded set of sensor readings3: Let $\Phi_{k}$ be a spatial fragmentation strategy4: $\Phi_{k}=\left\{\phi_{0},\phi_{1},\phi_{2},\ldots ,\phi_{n}\right\}$5: Let $\Omega_{k}$ be the frequency of aggregation▹ e.g., minutely, hourly, daily6: Let $\Sigma_{k}$ be a set of aggregate operations ▹ e.g., AVG, SUM, COUNT7: Create persistent storage for view $V_{\langle \hat{s},\Phi_{k},\Omega_{k}\rangle }$8: **for each** reading $r_{i}$ in S
**do**9:     $\left(\phi_{i},\tau_{i}\right)\leftarrow F\left(r_{i}\langle t_{i},x_{i},y_{i}\rangle \right);\;\phi_{i}\in \Phi_{k}$▹ Get the spatial fragment and temporal bin for $r_{i}$10:     $\sigma_{i}\leftarrow V_{\langle \hat{s},\Phi_{k},\Omega_{k}\rangle }\left(\phi_{i},\tau_{i}\right)$▹ Get the data summary $r_{i}$ should be aggregated into11:     **for each** operation AGGR in $\Sigma_{k}$
**do**▹ Update data summary aggregates12:         $\sigma_{\mathrm{AGGR}}\leftarrow \sigma_{i}\left[\mathrm{AGGR}\right]$13:         **if**
$\sigma_{\mathrm{AGGR}}=\emptyset$
**then**▹ If there is no aggregate for AGGR yet, then initialize it with $r_{i}$14:            $\sigma_{\mathrm{AGGR}}\leftarrow \mathrm{AGGR}\left(r_{i}\langle v_{i}\rangle \right)$15:         **else**▹ Otherwise, update the current aggregate for AGGR with $r_{i}$16:            Update $\sigma_{\mathrm{AGGR}}$ with $r_{i}\langle v_{i}\rangle$17:         **end if**18:         Update $\sigma_{\mathrm{AGGR}}$ in $\sigma_{i}$19:         Persist $\sigma_{i}$ in $V_{\langle \hat{s},\Phi_{k},\Omega_{k}\rangle }$▹ Finally, update the continuous view20:     **end for**21: **end for**

As soon as sensor observations start being ingested, Explora is capable of processing queries issued against the continuous views. The mechanism for query resolution varies from HS queries to ST queries. The subsections below detail the procedure for each category of queries, starting by defining their corresponding functions.

#### 3.4.2. Query Processing: *Historical-Spatial Queries*

Let us define HS—for *historical-spatial* queries—as a function that takes as inputs a specification of an arbitrary polygonal selection, $\phi_{q}$, from a 2-dimensional map (e.g., as an array of vertex coordinates) and optionally an interval of dates, $\tau_{q\langle \mathrm{start},\mathrm{end}\rangle }$, and delivers as output an array containing the data summaries aggregated over all the spatial fragments $\phi_{i}$ lying inside the perimeter defined by $\phi_{q}$, for all the temporal bins $\tau_{i}$ in $\tau_{q}$:

(4) $$\begin{array}{cc} \mathrm{HS}{|}_{V_{\langle s,\Phi ,\Omega \rangle }}:\left(\phi_{q},\tau_{q\langle \mathrm{start},\mathrm{end}\rangle }\right)\mapsto & \{\langle \tau_{m},\sigma_{m}\rangle ,\langle \tau_{m+1},\sigma_{m+1}\rangle ,\langle \tau_{m+2},\sigma_{m+2}\rangle ,\ldots ,\langle \tau_{n},\sigma_{n}\rangle \};\\ & \tau_{m}\geq \tau_{q\langle \mathrm{start}\rangle }\wedge \tau_{n}\leq \tau_{q\langle \mathrm{end}\rangle } \end{array}$$

where $V_{\langle s,\Phi ,\Omega \rangle }$ is the continuous view that the HS function is evaluated against and each $\sigma_{k}$ is the aggregated summary that results from combining the data summaries corresponding to the spatial fragments covered by $\phi_{q}$, for temporal bin $\tau_{k}$. The procedure for deriving said aggregated summaries is formally defined below in Algorithm 2. First, the set of spatial fragments $\Phi_{q}$ lying inside $\phi_{q}$ is computed (this operation has been represented as the *set intersection* in line 6). Then, the boundary temporal bins, $\tau_{m}$ and $\tau_{n}$, are defined by truncating the $\tau_{q\langle \mathrm{start}\rangle }$ and $\tau_{q\langle \mathrm{end}\rangle }$ dates, respectively, according to the frequency of aggregation $\Omega_{k}$ (lines 7 and 8). This is, for instance, if $\Omega_{k}$ is set to *hourly*, then the dates are truncated to the exact hour (e.g., 2019-09-22T12:47:32.767Z→2019-09-22T12:00:00.000Z). Once these time boundaries have been determined, the data summaries corresponding to the fragments in $\Phi_{q}$ are retrieved from the view and aggregated for each of the temporal bins in the interval [$\tau_{m}$, $\tau_{n}$] (lines 11–21). Finally, the resulting aggregated summaries, along with their corresponding temporal bins, are paired together and incrementally appended to the result set to assemble the summary time series returned as output ($R_{\mathrm{HS}}$) (lines 24–26).
**Algorithm 2** Explora query processing for *historical-spatial* queries.1: Let $V_{\langle \hat{s},\Phi_{k},\Omega_{k}\rangle }$ be a continuous view being fed with sensor observations from a variable $\hat{s}$, with spatial fragmentation $\Phi_{k}$ and aggregation frequency $\Omega_{k}$2: Let $\Sigma_{k}$ be a set of aggregate operations▹ e.g. AVG, SUM, COUNT3: **procedure**
HS ($\phi_{q},\tau_{q\langle \mathrm{start},\mathrm{end}\rangle }$)4:     **input:** an arbitraty polygonal selection from a 2D map ($\phi_{q}$) and a time interval ($\tau_{q\langle \mathrm{start},\mathrm{end}\rangle }$)5:     **output:** summary time-series ($R_{\mathrm{HS}}$)6:     $\Phi_{q}\leftarrow \Phi_{k}\cap \phi_{q}$▹ Get the set of spatial fragments inside $\phi_{q}$7:     $\tau_{m}\leftarrow {\mathrm{truncate}}_{\Omega_{k}}\left(\tau_{q\langle \mathrm{start}\rangle }\right)$▹ Starting temporal bin8:     $\tau_{n}\leftarrow {\mathrm{truncate}}_{\Omega_{k}}\left(\tau_{q\langle \mathrm{end}\rangle }\right)$▹ Ending temporal bin9:     $R_{\mathrm{HS}}\leftarrow \left\{\right\}$▹ Initialize result set10:     **for**
$\tau_{i}=\tau_{m}$**to**
$\tau_{n}$
**do**11:         $\sigma_{i}\leftarrow \left\{\right\}$▹ Initialize empty aggregated summary for $\tau_{i}$12:         **for each** fragment $\phi_{j}$ in $\Phi_{q}$
**do**13:            $\sigma_{j}\leftarrow V_{\langle \hat{s},\Phi_{k},\Omega_{k}\rangle }\left(\phi_{j},\tau_{i}\right)$▹ Get the data summary for $\phi_{j}$ and $\tau_{i}$14:            **for each** operation AGGR in $\Sigma_{k}$
**do**15:                $\sigma_{\mathrm{AGGR}}\leftarrow \sigma_{i}\left[\mathrm{AGGR}\right]$16:                **if**
$\sigma_{\mathrm{AGGR}}=\emptyset$
**then**▹ If there is no aggregate for AGGR yet, then initialize it with $\sigma_{j}$17:                    $\sigma_{\mathrm{AGGR}}\leftarrow \sigma_{j}\left[\mathrm{AGGR}\right]$18:                **else**▹ Otherwise, combine the existing aggregate for AGGR with $\sigma_{j}$19:                    Combine $\sigma_{\mathrm{AGGR}}$ with $\sigma_{j}\left[\mathrm{AGGR}\right]$20:                **end if**21:                Update $\sigma_{\mathrm{AGGR}}$ in $\sigma_{i}$▹ Update the aggregated summary $\sigma_{i}$22:            **end for**23:         **end for**24:         Append $\langle \tau_{i},\sigma_{i}\rangle$ to $R_{\mathrm{HS}}$25:     **end for**26:     **return**
$R_{\mathrm{HS}}$▹ The time series of aggregated summaries27: **end procedure**

#### 3.4.3. Query Processing: *Snapshot-Temporal Queries*

On the other hand, ST—for *snapshot-temporal* queries—is a function that takes as inputs the timestamp $\tau_{q}$ at which a snapshot of the state of the observed variable would be taken and optionally a polygonal selection, $\phi_{q}$, from a 2-dimensional map (if not provided, the snapshot would be computed over the entire region for which data is available). With these inputs, ST returns the collection of data summaries that correspond to the spatial fragments lying inside of $\phi_{q}$ (if provided) for the temporal bin $\tau_{x}$, where $\tau_{q}$ falls into:

(5) $$\begin{array}{cc} \mathrm{ST}{|}_{V_{\langle s,\Phi ,\Omega \rangle }}:\left(\tau_{q},\phi_{q}\right)\mapsto & \{\langle \phi_{a},\sigma_{a}\rangle ,\langle \phi_{b},\sigma_{b}\rangle ,\langle \phi_{c},\sigma_{c}\rangle ,\ldots \};\\ & \phi_{x}\in \Phi \cap \phi_{q} \end{array}$$

where $V_{\langle s,\Phi ,\Omega \rangle }$ is the continuous view that the ST function is evaluated against and each $\sigma_{x}$ is a data summary registered under the temporal bin $\tau_{x}$ (namely, the one $\tau_{q}$ fits into). The generic sequence of steps followed by this function is detailed below in Algorithm 3. The procedure is similar to the one defined for the HS function. It also starts by computing the set of spatial fragments lying under the selected polygonal region and by determining $\tau_{x}$ from the provided timestamp ($\tau_{q}$) by applying a truncate operation (lines 5 and 6). Then, the data summaries available in the view are filtered, so that only those corresponding to the spatial fragments covered by $\phi_{q}$ and registered under $\tau_{x}$ are retrieved (lines 8–14). These summaries and their corresponding spatial fragments are coupled together and appended to a collection of tuples ($R_{\mathrm{ST}}$), representing a temporal snapshot of the observed variable.
**Algorithm 3** Explora query processing for *snapshot-temporal* queries.1: Let $V_{\langle \hat{s},\Phi_{k},\Omega_{k}\rangle }$ be a continuous view being fed with sensor observations from a variable $\hat{s}$, with spatial fragmentation $\Phi_{k}$ and aggregation frequency $\Omega_{k}$2: **procedure**
TS ($\tau_{q},\phi_{q}$)3:     **input:** a snapshot timestamp ($\tau_{q}$) and a polygonal selection from a 2D map ($\phi_{q}$)4:     **output:** temporal snapshot ($R_{\mathrm{ST}}$)5:     $\Phi_{q}\leftarrow \Phi_{k}\cap \phi_{q}$▹ Get the set of spatial fragments inside $\phi_{q}$6:     $\tau_{x}\leftarrow {\mathrm{truncate}}_{\Omega_{k}}\left(\tau_{q}\right)$▹ Get the querying temporal bin7:     $R_{\mathrm{ST}}\leftarrow \left\{\right\}$▹ Initialize result set8:     **for each** fragment $\phi_{x}$ in $\Phi_{q}$
**do**9:         $\sigma_{x}\leftarrow V_{\langle \hat{s},\Phi_{k},\Omega_{k}\rangle }\left(\phi_{x},\tau_{x}\right)$▹ Get the data summary for $\phi_{x}$ and $\tau_{x}$10:         **if**
$\sigma_{x}\neq \emptyset$**then**▹ If there is a data summary under $\left(\phi_{x},\tau_{x}\right)$11:            Append $\langle \phi_{x},\sigma_{x}\rangle$ to $R_{\mathrm{ST}}$12:         **end if**13:     **end for**14:     **return**
$R_{\mathrm{ST}}$▹ Snapshot of $\hat{s}$, over $\phi_{q}$ at $\tau_{x}$15: **end procedure**

The three algorithms formulated in this section lie at the core of Explora, allowing for interactive exploration of mobile sensor data. It is worth noting that, since these algorithms operate on discretized units of space and time, in most of the cases, they would only manage to deliver approximate query answers; this is, the gain in speed this framework brings in entails a loss in accuracy. Nevertheless, for use cases in visual exploratory analysis, these estimates are able to provide relevant insights on the state and historical behaviour of the observed variables. Later, in Section 5, a metric is introduced to measure accuracy of queries issued against continuous views—under several spatiotemporal fragmentation strategies—w.r.t. queries running on the base raw data.

To recap, this section developed a thorough description of the framework devised for enabling interactive exploration of mobile sensor data in smart cities. It started by identifying the framework requirements and features. Then, a description of the key techniques behind the formulation of the proposed framework was discussed. Next, the definition of the layered architecture adopted for the proposed framework along with the description of its constituent modules were addressed, and finally, a comprehensive presentation of the mechanisms behind the stream processing pipeline that enables the continuous generation of data synopsis structures as well as the procedures defined to speed up spatiotemporal queries, which profit from said data synopsis structures, were made.

## 4. Prototype Implementation

This section explores the applicability of the Explora framework for enabling interactive exploration of live and historical smart city data by harnessing existing open source data technologies. First, an application scenario within the context of mobile sensor data in smart cities is described. Then, three target use cases of visual exploratory applications are defined, incorporating the *elementary* and *general* exploratory tasks identified in the previous section to provide more elaborate interaction workflows. Lastly, two implementations aimed at supporting the defined use cases are detailed, one based on a traditional spatial time-series database approach and another using a distributed stream processing approach.

### 4.1. Application Scenario: The Bel-Air Project

**Figure 7:**
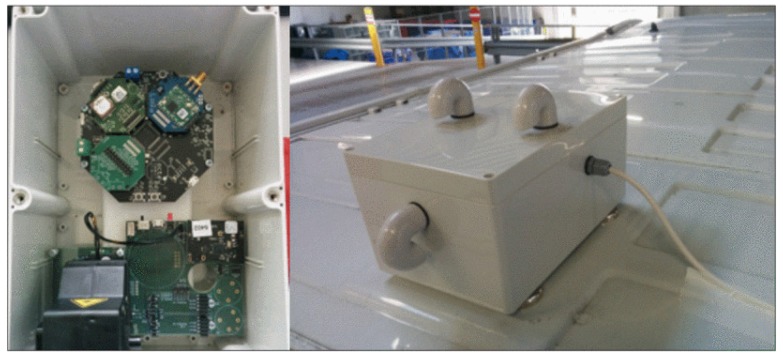
Setup of air quality sensors installed on the roofs of the *Bpost* delivery vans as part of the *Bel-Air* project.

The *Bel-Air* project is part of the *City of Things* (CoT) [46] initiative that is being implemented in the city of Antwerp, Belgium, in a joint effort that involves businesses, government, and academia. This initiative aims at putting together a city living lab and technical testbed environment, which allows researchers and developers to easily set up and validate IoT experiments. Within CoT, the Bel-Air project is particularly concerned with finding efficient mechanisms to accurately measure the air quality over the city. Since the costs of rolling out a dense network of fixed sensors across a large urban area could be prohibitively expensive, the Bel-Air project established a partnership with the Belgian Postal service (*Bpost*) to attach highly sensitive sensors to the roofs of the mail delivery vans that traverse the city on a daily basis (see Figure 7). These sensors conduct periodic measurements on environmental variables such as temperature, humidity, and air pollution (particulate matter, nitrogen dioxide, etc.), which are timestamped and geotagged before being sent over the network to a persistent storage. This mobile sensor setup together with some additional sensors deployed at fixed locations allow mapping the air quality of the entire city of Antwerp in a cost-effective way.

Efficient mechanisms for visual exploratory analysis over the data delivered by the mobile-sensor setup of the Bel-Air project can help get relevant insights regarding the status of air quality across the urban area of Antwerp, which would further allow to timely take the proper course of action to mitigate the problems caused by elevated levels of pollution. This scenario serves as the context for a proof-of-concept realization for the proposed framework. The next section describes a number of target use cases to test the applicability of the Explora approach.

### 4.2. Target Use Cases for Visual Exploratory Applications on Spatiotemporal Data

#### 4.2.1. Visualizing the Temporal Change of an Observed Variable over a Certain Region

**Figure 8:**
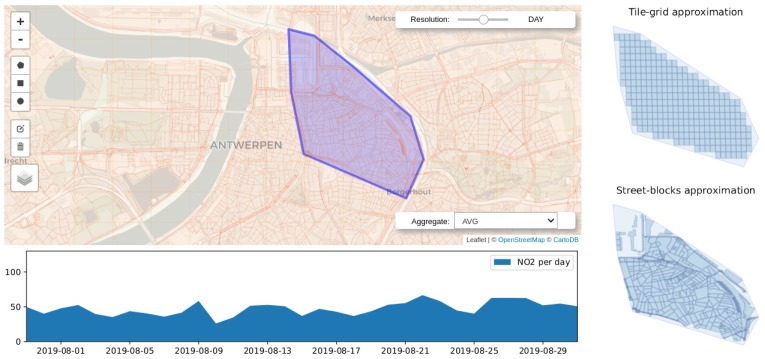
Application allowing users to examine the change over time of an air quality measure on a certain geospatial region: Notice at the right hand side how tiles and street-blocks would approximate the area of the provided polygonal selection.

This use case has to do with allowing users to pose visual queries aimed at examining the historical behaviour of an air quality variable by defining a polygonal selection on a 2-dimensional map. Queries are further parameterized, allowing users to specify traits such as the *aggregate function* they want to be applied on the data, the *time resolution* (per-minute, per-hour, or per-day) or *time period* (last 5 minutes, last hour, etc.) they want the results to be displayed on, and whether the query should be issued against the raw sensor data or run against the continuous views computed during data ingestion. Figure 2 (in Section 3.2.1) and Figure 8 below portray examples of this use case.

#### 4.2.2. Progressive Approximate Query Answering

**Figure 9:**
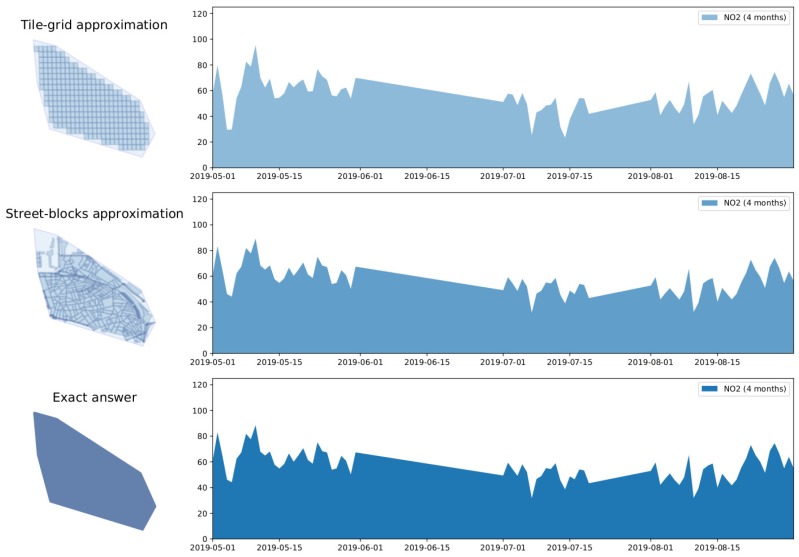
Progressive approximate query answering: The approximate time series at the top gets gradually refined until the exact answer is presented to the user.

Aiming at improving the user experience in terms of perceived responsiveness, queries supporting visual exploratory actions can profit from the reduced latency expected from synopsis data structures. In this sense, users can be presented first with an approximate answer to their requests, which then is continuously refined as time goes on, until the exact result—computed on the raw data—is finally displayed. To this end, multiple continuous views are required to be computed during data ingestion, featuring progressively finer geospatial resolution. Consider for instance the time series charts in Figure 9, corresponding to the polygonal selection in Figure 8 over a period of 4 months. Notice how the resulting time series is progressively refined from the chart at the top to the one at the bottom, which corresponds to the final exact answer derived from the raw sensor observations.

#### 4.2.3. Dynamic Choropleth Map

This use case concerns the visualization of the historical behaviour of a given variable, this time by displaying a sequence of successive temporal snapshots and by allowing the user to transition between them on command using interactive controls (e.g., back and forward buttons or a time slider). An example of this use case was presented earlier in Figure 3 when discussing the execution of a *general* exploratory task as a composite of multiple *elementary* tasks.

### 4.3. Proof-of-Concept Implementations of *Explora*

**Figure 10:**
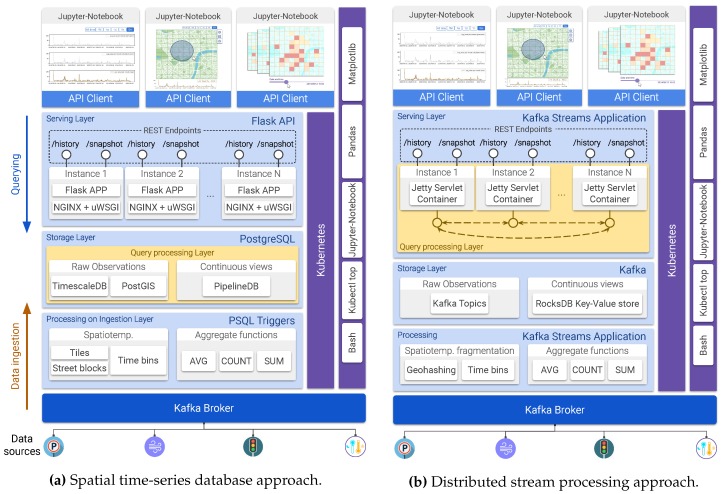
Proof-of-concept implementations of Explora.

This section describes two realizations of the *Explora* framework: the first one harnesses a series of extensions of the *PostgreSQL* open-source relational database management system (RDBMS), which endow this database engine with capabilities for efficiently storing and indexing time-series and geospatial data. The second implementation draws on the distributed stream processing engine provided by Apache Kafka [47] to process the feed of sensor observations from the Bel-Air project setup. Figure 10 presents two diagrams mapping the technologies used in both implementations to each of the tiers and components of the Explora framework. Let us first consider the modules which are common to both implementations and then proceed to a detailed description of those that are specific to each approach.

**Figure 11:**
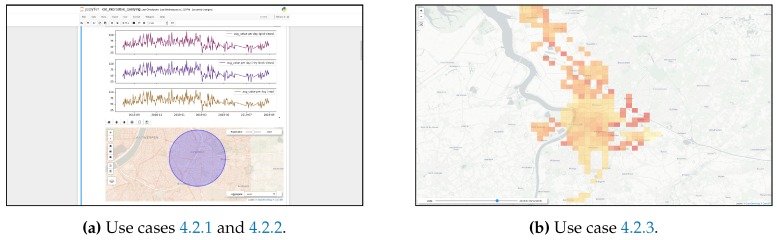
Screen captures of the Jupyter notebooks implementing the target use cases defined in Section 4.2.

**Event** **log** *Apache Kafka* is used to implement this layer of the architecture. Kafka provides a number of tools for processing and analyzing streams of data, including a distributed message broker that adopts the *publish–subscribe* pattern. This Kafka broker allows for registering each of the incoming sensor observations into a partitioned append-only log, maintaining them over a fixed configurable retention period, which enable multiple consumers (as many as the number of partitions) to read and process the collected data in an asynchronous-concurrent way.
**Container** **orchestration** The components in the *serving*, *storage*, and *processing on ingestion* layers are built as Docker containers and are deployed on a Kubernetes cluster, consisting of one master node and three working nodes, all of them running *Ubuntu 18.04.3 LTS*.
**Performance** **monitoring** Data regarding query response time, query accuracy, and computing resources usage for all the components of the system is captured via bash and Python scripting. Once collected, this information is analyzed and visualized through a series of *Jupyter notebooks* that make use of the *Pandas* and *Matplotlib* Python libraries.
**Serving** **Layer** A REST API is implemented for serving client applications. In the PostgreSQL-based implementation (see Figure 10a), this API is provided by using the *Flask* web framework for Python and *NGINX*+*uWSGI* as an application server, while in the distributed stream processing approach (Figure 10b), this API runs on a *Jetty servlet container*. This REST API consists of two endpoints: one for handling *historical-spatial* queries and the other for *snapshot-temporal* queries. The specification of each of the API endpoints is presented below in Table 1. Multiple instances of the API server are deployed to balance the load and to provide high availability.
**Client** **applications** Two Jupyter notebooks are deployed as client applications, one implementing the first two use cases described in Section 4.2 and another implementing the third use case. Figure 11 shows screen captures taken from these implementations.
  These notebooks consume the API available in the serving layer to resolve the *historical-spatial* and *snapshot-temporal* queries that support the interaction with end users.

#### 4.3.1. Spatial Time-Series Database Approach

**Processing** **on** **ingestion** PostgreSQL triggers are used to implement the ingestion procedure described in Algorithm 1. These trigger functions are invoked for each of the sensor readings being consumed from the *Kafka broker*, relaying them to the corresponding continuous views for aggregation before being persisted into the time-series storage. Two spatial fragmentation schemas have been laid over the region covered by the mobile sensors, namely a *tile grid* built according to the *geohash* encoding algorithm by Niemeyer G. [48] (see Figure 1a for reference) and a grid corresponding to the *street-blocks* of the city of Antwerp (see Figure 1b). Additionally, four aggregation frequencies were considered, fragmenting time into minutely, hourly, daily, and monthly bins. In consequence, under this setup, eight continuous views (2-spatial fragmentation schemas × 4-aggregation frequencies) are computed, holding data summaries that comprise the results of three aggregate functions applied over the incoming stream of sensor observations: the arithmetic average of the measured values (AVG), the sum of the measurements (SUM), and number of reported readings (COUNT).
**Storage** **and** **query** **processing** **layer** For these layers, three open-source extensions of PostgreSQL are set up on top of this database engine, enabling it to store and query time-series data, to support geospatial operations, and to incrementally create and persist continuous views:
  - *TimescaleDB* [49] is a time-series database working on top of PostgreSQL, thus being able to offer a *full SQL* querying interface while supporting fast data ingestion. Raw sensor readings consumed from the *Kafka broker* are formatted and stored into a TimescaleDB *Hypertable*, which partitions data in the temporal dimension for efficient ingestion and fast retrieval.
  - *PostGIS* [50] is a spatial extension that allows PostgreSQL to store and query information about location and mapping. With PostGIS in place, the GeoJSON specifications of the *tile* and *street-block* grids are stored as two spatial tables, for which the records correspond to individual tile/street-block from the spatial fragmentation schemes. Likewise, each one of the records from the TimescaleDB Hypertable are augmented with a PostGIS geography object that corresponds to the sensor reading location. This enables the execution of spatial join operations required later during the querying stage to address calculations such as *point-in-polygon* and *polygon intersection*.
  - *PipelineDB* [51] is an extension that enables the computation of continuous aggregates on time-series data, storing the results into regular PostgreSQL tables. The eight continuous views mentioned earlier are created in PipelineDB and incrementally computed as continuous queries running against the stream of sensor observations being handed in through the trigger functions in the ingestion layer. For illustration, Listing 1 presents the SQL statement used in PipelineDB for creating a view that computes the three stated aggregates on a per-minute basis.

Listing 1: Example of a view creation statement in PipelineDB.
CREATE VIEW aq_no2_minutely_view WITH (action = materialize) AS
  SELECT fragment_id, observed_var, minute (time) AS ts,    COUNT (*) AS count,    SUM (value) AS sum_value,    AVG (value) AS avg_value  FROM aq_no2_stream -- *stream of NO2 sensor measurements*  GROUP BY fragment_id, observed_var, ts;


Since PostgreSQL is the underlying storage technology used in this setup, it is possible to translate the procedures for handling *historical-spatial* and *snapshot-temporal* queries (from Algorithms 2 and 3, respectively) into declarative SQL statements, leveraging the expressiveness of this language along with the capabilities of the implemented extensions. An example of said statements is presented below in Listing 2.
Listing 2: Example of a HS query statement running on PipelineDB.SELECT observed_var, ts, combine (avg_value) AS avg_valueFROM aq_no2_minutely_viewINNER JOIN tile_grid ON aq_no2_minutely_view. fragment_id = tile_grid.idWHERE ST_Contains (ST_GeomFromText (’<QUERY_POLYGON>’), tile_grid.geom)GROUP BY observed_var, tsORDER BY ts;-- *QUERY_POLYGON: Well - Known Text (WKT) representation*-- *of the user’s polygonal selection*.

#### 4.3.2. Distributed Stream Processing Approach

**Processing** **on** **ingestion** A *Kafka streams* application is implemented for this layer, according to the procedure in Algorithm 1. The Kafka streams library provides an API for conducting distributed stateful transformations on the feed of sensor observations being pushed to the *Kafka broker* by enabling multiple stream processor instances to consume the partitioned Kafka topics that the sensor readings are being written to. In consequence, the global application state is also partitioned into a distributed key-value store, instances of which are collocated with the working stream processors. Since Kafka streams does not support spatial operations out-of-the-box, in order to set up a statiotemporal fragmentation schema, a compound record key was associated to each of the incoming sensor observations, consisting of their *geohash* code (a base 32 sequence of 12 characters encoding the latitude and longitude of the measurement), along with their correponding timestamp, formatted as in the example shown below:
  $$\overset{\overset{\mathrm{geohash}}{⏞}}{\underset{\underset{\left\{\mathrm{lat}:51.012818,\;\mathrm{lon}:3.707970\right\}}{⏟}}{u14\mathrm{dhqs}4\mathrm{cpbp}}}\#\overset{\mathrm{timestamp}}{\overbrace{\underset{\mathrm{date}:\;2019/11/01}{\underbrace{20191101}}:\underset{\mathrm{time}:\;14:31:15}{\underbrace{143115}}:\underset{\mathrm{milliseconds}}{\underbrace{344}}}}$$

By augmenting sensor observations with keys structured in this way, the implemented ingestion procedure is able to set up a geohash-based spatial grid, leveraging the fact that readings sharing the first *k*-geohash characters fall into the same geospatial region identified by such *k*-character prefix. Likewise, the same procedure uses timestamp prefixes to set up a time-partitioning layout over the incoming stream of sensor readings, pushing records into minutely, hourly, daily, and monthly bins. Thereafter, data summaries are continuously computed on each of the geohash-based spatial fragments for each of the time partitions, and their results are persisted into the distributed state store. As an illustration, Listing 3 presents an example of the continuous views generated by the Kafka streams application.
Listing 3: Example of a continuous view with hourly time bins in *Kafka Streams*. The segment presented corresponds to the spatial fragment identified by the geohash prefix u14dhq.…u14dhq #20191101:140000:000: {AVG: 54.32, SUM: 182678.16, COUNT:3363},u14dhq #20191101:150000:000: {AVG: 32.10, SUM: 111964.80, COUNT:3488},u14dhq #20191101:160000:000: {AVG: 45.13, SUM: 147755.62, COUNT:3274},u14dhq #20191101:170000:000: {AVG: 90.08, SUM: 304560.48, COUNT:3381},…

**Figure 12:**
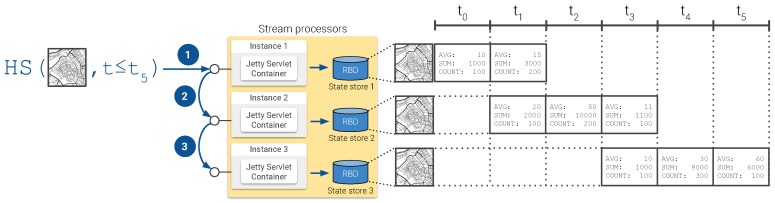
Procedure for distributed query resolution.

**Storage** **layer** This layer is also supported by tools provided by Kafka: raw sensor observations are stored into Kafka topics, while continuous views generated in the ingestion layer are stored into a distributed key-value database known as RocksDB [52], which Kafka uses as the default state store for stream applications. While records stored in Kafka topics are not directly queryable, continuous views in RocksDB allow simple key-based lookup and range queries. This is why a major part of the query processing needs to be conducted in the serving layer, when handling the client application requests.
**Query** **processing** **and** **serving** **layer** In this distributed setup, an instance of the REST API serving client requests is hosted on each of the Kafka stream processors. Each of these instances is only capable of answering queries on the portion of the application state available to the hosting stream processor. Therefore, resolving a query on the global state requires combining the results computed on the state available to each of the stream processor thus far. Consider for instance the example presented in Figure 12, illustrating the procedure for a setup with three stream processors, resolving a *historical-spatial* query: (1) The query reaches one of the instances of the serving layer API. This instance processes the query against the version of the continuous view persisted on its own state store. (2) Then, the query is relayed to a second instance to retrieve the data summaries from its corresponding state store and to combine them with those obtained from the first instance. (3) This process is repeated until the query reaches the last API instance. Finally, the resulting sequence of aggregated data summaries is retrieved to the client application.
  It is worth noting that the simplicity of the querying interface offered by the state stores—limited basically to key-based lookups and range queries—along with the key-value data model they adopt pay off in terms of query processing time, as will be shown when discussing the performance of these proof-of-concept implementations in the following section.

## 5. Experimental Evaluation

The previous section explored two proof-of-concept implementations of the Explora framework, proving its ability to support typical use cases for visual exploratory applications on mobile sensor data. This section addresses a performance evaluation conducted on both implementations on a feed of air quality sensor observations collected from the *Bel-Air* smart city setup.

### 5.1. Query Accuracy Metric

The performance evaluation reported herein is mainly focused on determining to what extent the continuous computation of data summaries applied in Explora effectively reduces the query response time on spatiotemporal data and what is the cost of such increase in responsiveness in terms of query accuracy. The latter was determined by defining a metric accounting for the average distance between the elements of the result sets obtained when querying continuous views—i.e., approximate answer—against those retrieved when querying the base raw sensor data—i.e., exact answer. Let $X_{q}$ and $Y_{q}$ be two result sets obtained from running a query *q* against both a continuous view V and the base sensor data R, respectively. $X_{q}$ and $Y_{q}$ can be regarded as *relations* since they designate a set of ordered pairs:

(6) $$\begin{array}{cc} X_{q}= & \left\{\langle k_{x1},v_{x1}\rangle ,\langle k_{x2},v_{x2}\rangle ,\ldots ,\langle k_{xm},v_{xm}\rangle \right\}\\ Y_{q}= & \left\{\langle k_{y1},v_{y1}\rangle ,\langle k_{y2},v_{y2}\rangle ,\ldots ,\langle k_{yn},v_{yn}\rangle \right\} \end{array}$$

With $k_{xi}$ and $k_{yi}$ being spatial-fragment identifiers or timestamps and $v_{xi}$ and $v_{yi}$ being aggregate values. Ideally, $X_{q}$ and $Y_{q}$ should have the exact same set of keys and values; this is, the distance between them (tuple-wise) should be zero. However, due to the applied spatiotemporal fragmentation scheme, data summaries—upon which queries are resolved—can only match spatial and temporal query predicates in an approximate manner. In consequence, key-value sets might differ from $X_{q}$ to $Y_{q}$. To estimate the average tuple-wise distance—henceforth, *distance*—between these two result sets, first, a *full outer-join* operation is computed:

(7) $$\begin{array}{cc} Z_{q} & =X_{q}⟗Y_{q}\\ & =\{\langle k_{z1},(v_{xz1},v_{yz1})\rangle ,\langle k_{z2},(v_{xz2},v_{yz2})\rangle ,\langle k_{z3},(v_{xz3},v_{yz3})\rangle ,\ldots \}\\ & v_{xzi}=0,\;\mathrm{if}\;k_{zi}\notin X_{q}\wedge v_{yzi}=0\;\mathrm{if}\;k_{zi}\notin Y_{q} \end{array}$$

Then, the distance (*d*) between these two result sets is estimated as follows:

(8) $$\begin{array}{c} d:X_{q}\times Y_{q}\mapsto [0,1],\\ d\left(X_{q},Y_{q}\right)=\frac{1}{|Z_{q}|}\sum_{i}^{|Z_{q}|}\frac{|v_{xzi}-v_{yzi}|}{|v_{xzi}\left|+\right|v_{yzi}|} \end{array}$$

where $|Z_{q}|$ denotes the cardinality of the set resulting from the outer-join operation in Equation (7). One appealing feature of this distance metric is that it provides a normalized symmetrical measure of the dissimilarity between two result sets, which makes it more easily interpretable than alternative distance metrics such as *dynamic time warping* (DTW) [53] used for measuring the similarity between two temporal sequences.

### 5.2. Experimental Setup

The data set collected for this performance evaluation covers about one-year’s worth of sensor measurements (from August 2018 to August 2019) made to map the situation of air pollutant emissions over the city of Antwerp. The two proof-of-concept setups detailed in Section 4 were deployed to a Kubernetes cluster consisting of one master and three worker nodes, set up on the *imec/IDLab Virtual Wall* environment [54]. Table 2 lists the versions of the software tools used in these implementations.

The process for collecting performance information on both proof-of-concept setups started by recording the queries generated during one user session on the first setup. This collection of queries—designated henceforth as *workload*—amounts to 222 statements comprising a wide range of query predicates (polygonal selections, timestamps, time intervals, etc.), 64% of which correspond to *historical-spatial* queries while the remaining 36% are *snapshot-temporal* requests. The collection of historical-spatial queries can be further divided into statements with a predicate in the temporal dimension (i.e., those querying over a certain period of time provided by the user) and queries without said predicate (namely, those querying over the whole period of available data thus far). Table 3 shows the final composition of the query workload, considering the discussed classification.

To determine how each of the Explora implementations performs as the amount of ingested data increases, the test air quality data was fed to both setups in batches of one-month’s worth of data. This way, at the end of each batch increment, the sequence of request included in the workload was run on both implementations while monitoring query response time and query accuracy. Each batch of raw sensor data was ingested and aggregated into a geohash-based tile grid (for which precision was set to a six-character geohash prefix) and—only for the spatial time-series database setup—a street-blocks based grid, which partitions the urban area of Antwerp into 12.230 polygonal regions.

### 5.3. Results

#### 5.3.1. Continuous Views Storage Footprint

**Figure 13:**
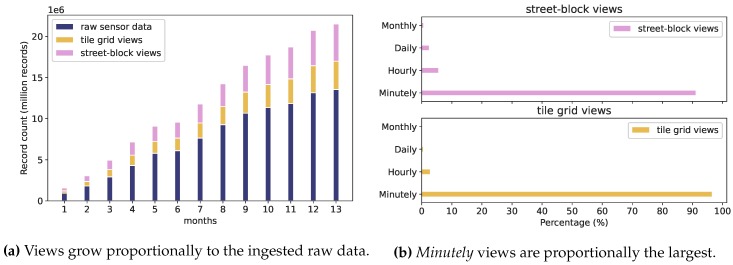
Storage footprint of continuous views.

The continuous views generated through Explora are by definition redundant data structures for read optimization [55] and, as such, entail a storage overhead. In this sense, Figure 13a illustrates the proportion of the number of records (i.e., data summaries) registered in the views w.r.t. the total count of raw sensor observations ingested per month for both tile and street-block grids. On average, tile grid views and street-block views amount, respectively, to 26.5% and 33.8% of the total record count for sensor readings. Since street-block views rely on a finer (and irregular) spatial fragmentation strategy than that used for tile views, the number of data summaries placed into the former views is larger in proportion to the amount of raw sensor observations.

In the same vein, Figure 13b shows that most of the storage overhead is due to views with aggregation frequency set to one minute, accounting—in both tile and street-block views—for more than 90% of the total amount of generated data summaries. Again, as stated earlier in Section 3.2.2, the lower the resolution of the spatiotemporal fragmentation scheme, the smaller the size of the corresponding view: while for one-year’s worth of sensor data, there might be around 8.640 *hourly* data summaries per spatial fragment, the corresponding *minutely* summaries would amount to 518.400, which explains the stark difference between the *minutely* view proportions and the second-largest *hourly* views.

#### 5.3.2. Query Response Time for HS Queries without Time Predicate

**Figure 14:**
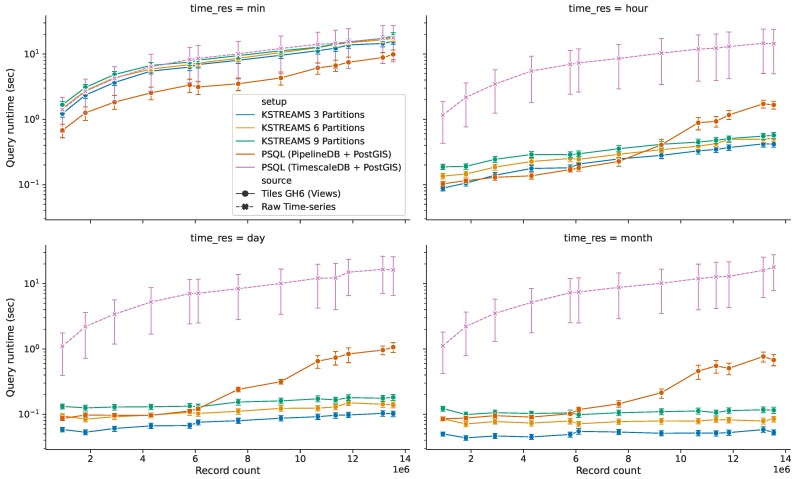
Query response time vs. volume of ingested data: HS queries without time predicate.

When the serving API receives a *historical-spatial* request providing only the spatial parameter (and no conditions on the temporal dimension), the corresponding response is computed over the full extent of data available thus far. The response time reported for this kind of queries with regards to the amount of ingested sensor readings is illustrated in Figure 14. Results from multiple setups are presented in these charts in order to compare both implementations of Explora. For the *spatial time-series database* approach (PostgreSQL based), the query response time on the raw sensor observations (*TimescaleDB + PostGIS*) and continuous views (*PipelineDB + PostGIS*) are reported; while for the *distributed stream processing approach* (Kafka based), results obtained from running Apache Kafka with three different partition settings are presented: 3 partitions/3 stream processors (*KSTREAMS 3 Partitions*), 6 partitions/6 stream processors (*KSTREAMS 6 Partitions*), and 9 partitions/9 stream processors (*KSTREAMS 9 Partitions*). Query processing time from the *TimescaleDB + PostGIS* setup serves as reference to estimate the performance gain in query response time for the remaining setups. These time measurements were conducted along the four considered temporal resolutions, namely *per-minute*, *per-hour*, *per-day*, and *per-month* bins. In light of the results obtained, it is worth highlighting four key facts:

(*i*) Query response time on the raw data (dashed line in Figure 14) behaves nearly the same along the four temporal resolutions, displaying a linear increase as the amount of data ingested grows larger. This describes an expected system’s response, since each of these queries involves running expensive *sequential scan* operations over the full collection of raw sensor readings. This way, response time for these requests increases proportional to the amount of ingested sensor observations, regardless of the requested temporal resolution.
(*ii*) Continuous views (solid lines in Figure 14) in general outperform the base raw data for both implementations of Explora. Only for views with per-minute temporal bins the performance benefit from using these synopsis structures is compromised due to the considerable size of said structures relative to the raw data (and to the remaining views, as evidenced earlier in Figure 13). However, even in this case, queries perform 1.1–1.3× faster in the *3-partition/3-processors* Kafka setup and 1.8–2.9× faster in the *PipelineDB + PostGIS* setup compared to queries running against the raw data. For the other considered time resolutions, queries running on the corresponding views perform up to two orders of magnitude faster than the reference setup, reaching sub-second response times in all cases.
(*iii*) When it comes to distributed stream processing, increasing parallelism—i.e., adding partitions and stream processors accordingly—actually leads to a slight decline in performance, which can be attributed to the overhead due to the process of combining the partial aggregates computed on each of the stream processors, which also implies data exchange among said processors (network overhead). That said, this approach still delivers a more stable response as the data volume grows compared to the spatial time-series approach, describing a linear-time trend for which the slope tends to zero as the temporal resolution of the aggregates decreases—notice the almost constant time for views with per-month temporal bins.
(*iv*) For ingested data under 6–8 million sensor observations, queries on the spatial time-series approach either outperform or closely follow the performance of those from distributed streaming setups. From 8 million records onwards, the query response time for the *PipelineDB + PostGIS* setup branches out, describing an exponential growth. In this situation, given the increased volume of data, indexed tables can no longer fit in the available memory; in consequence, parts of the index are repeatedly swap in and out of the database buffer pool, leading to a performance degradation.

#### 5.3.3. Query Response Time for HS Queries with Time Predicate

**Figure 15:**
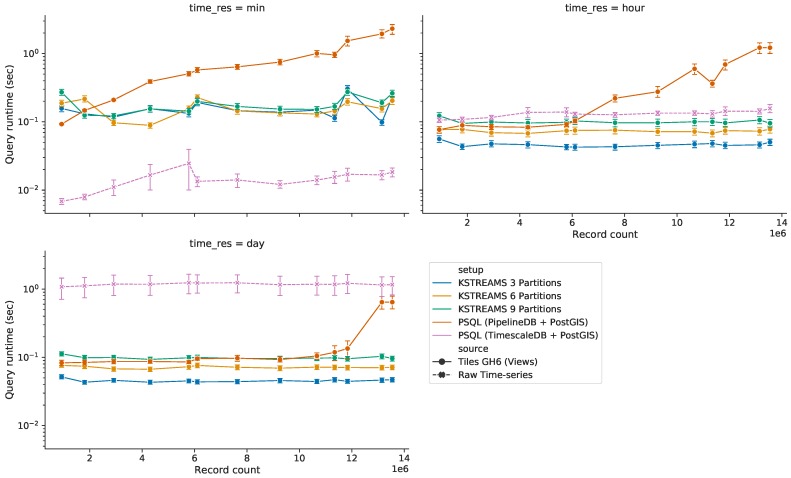
Query response time vs. volume of ingested data: HS queries with time predicate.

This part of the evaluation deals with a more practical and sensible kind of query, namely those with predicates in both spatial and time dimensions. Figure 15 describes the performance for queries running on the six considered setups for five predefined time intervals: *last 5 minutes* and *last hour* running on *minutely* views; *last day* running on *hourly* views; and *last week* and *last month* running on *daily* views.

The obtained results show how the reference setup (*TimescaleDB + PostGIS*) is able to deliver almost constant-time performance for queries requesting *hourly* and *daily* time resolutions and outperforms the alternative implementations based on synopsis data structures with *minutely* time bins. This behaviour stems from TimescaleDB taking advantage of the inherent time-ordering of the ingested sensor observations to only process the most recent data. On the other hand, once again, the distributed stream processing approach stands out as the system with the most stable performance, featuring a nearly constant-time response as the amount of ingested data increases and sub-second query latency for all the considered time intervals. Meanwhile, the performance of the spatial time-series database (*PipelineBD + PostGIS*) approach falls behind, as it struggles to deliver a consistent time response as data grows larger.

#### 5.3.4. Query Response Time for ST Queries

**Figure 16:**
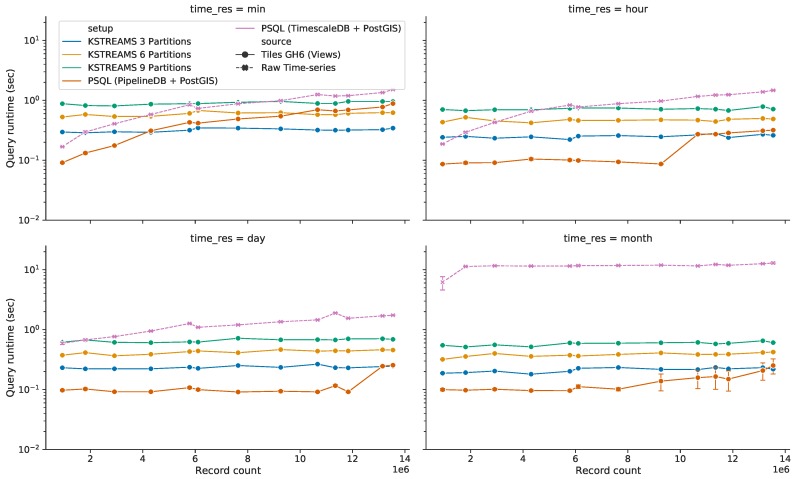
Query response time vs. volume of ingested data: ST queries.

*Snapshot-temporal* queries provide a time-slice visual of the status of the observed variable over the geospatial region being displayed on the user’s screen for a given timestamp and for a specific time resolution determining the span of time covered in the query computation (i.e., *one minute*, *one hour*, *one day*, or *one month*). Figure 16 below reports on the performance for this kind of query as the amount of data ingested increases.

According to this test, the distributed stream processing setups deliver a constant-time response as data volume grows for all considered time resolutions in contrast to the alternative PostgreSQL setups, for which response is affected by the amount of data available (notice the linear-time performance for queries running with *one minute* time resolution) and the temporal interval over which the query is computed (notice how, for the reference setup, query latency tends to increase as this interval goes from *one minute* to one *one month*). This behaviour obeys to the fact that the distributed key-value database storing the partitioned continuous views enables constant-time key-based lookups, making the procedure implemented for resolving *snapshot-temporal* queries independent of the amount of data available and only subject to the size of the visible (or selected) geospatial region. Another significant result from this test is that, overall, both implementations of the Explora framework deliver sub-second response times, proving these approaches effective to enable interactive-level performance for *snapshot-temporal* queries.

#### 5.3.5. Query Accuracy on Continuous Views

**Figure 17:**
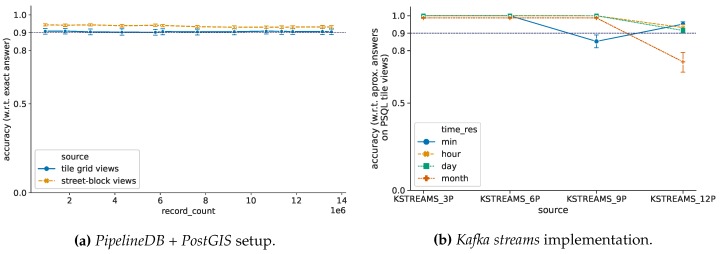
Query accuracy on the continuous views computed with Explora: (**a**) Accuracy on both tile-grid and street-block views is above 90% on average. (**b**) Accuracy in relation to approximate answers from the *PipelineDB + PostGIS* setup: increasing the number of partitions eventually compromises query accuracy.

The main caveat of using synopsis data structures for answering spatiotemporal queries is their inherent loss in accuracy. Figure 17 illustrates the level of accuracy attained in both of the proof-of-concept implementations of Explora for different setups. In these charts, accuracy is defined as the complement of the distance metric formulated earlier in Section 5.1:

$$accuracy=1-d(X_{q},Y_{q})$$

where $X_{q}$ is a result set obtained upon running a query *q* on one of the available continuous views and $Y_{q}$ is a reference result set. For the spatial time-series database implementation, Figure 17a portrays the accuracy achieved for queries running on tile-based and street-block based views, as a function of the amount of data ingested. In this case, the reference result sets are those computed on the raw sensor observations for each query in the test workload. According to these results, the cost incurred in terms of accuracy is, on average, less than 10% for both types of views. It is also clear from the chart how using a finer spatial fragmentation schema allows for a more accurate approximation: queries running on the street-block views are 3.64% closer to the exact answer than those running on the coarser tile-based views.

On the other hand, for the distributed stream processing implementation, query accuracy is measured as a function of the number of partitions (and stream processors) used to split the ingested data and to generate the distributed continuous views under the premise that increasing parallelism implies increased error probability. Since views from this setup are based on the same geohash tiles from the *PipelineDB + PostGIS* implementation, the query accuracy obtained in said implementation defines an upper bound for the distributed processing approach in this particular setup. That is why query accuracy in Figure 17b is estimated in relation to the approximate answers derived from the tile views of the PostgreSQL-based setup. The reported results indicate an effective drop in the expected accuracy once data and processing are split up into more that six partitions, evidencing that increased parallelism not only impacts query latency but also can eventually compromise the accuracy of the answers computed on continuous views.

## 6. Conclusions

Supporting visual-interactive exploration on top of the massive volumes of smart city data being generated nowadays remains largely an open problem. The stringent latency requirements typical of these kind of applications call for proactive and flexible data management mechanisms able to serve users with prompt answers to their information requirements, based on the most recent data available. In this sense, this paper introduced Explora, a microservice-based data management framework for spatiotemporal data produced in smart city environments (*i*) that leverages stream processing methods to continuously compute synopsis data structures over the live feed of measurements coming from mobile sensor, (*ii*) that defines a uniform interface to query said structures based on recurrent user interaction patterns, and (*iii*) that monitors system and query performance.

The experimental evaluation conducted on two proof-of-concept implementation of Explora—one based on a traditional spatial time-series database approach and another using a distributed stream processing pipeline—proved the feasibility of the proposed framework, being able to serve expensive spatiotemporal queries with sub-second performance over a continuously increasing amount of sensor data (reaching up to 2 orders of magnitude speedup in comparison to queries running on the base raw observations) at the expense of less than 10% loss in accuracy and around 30% of storage overhead.

A current limitation of the Explora framework is that the set of aggregate operations used for building the continuous synopsis structures (e.g., *average*, *sum*, and *count* in the described implementations) has to be defined upfront. In this sense, future work on this research will extend the framework to incorporate a *pluggable* mechanism that enables developers/users to provide custom aggregates as extensions that would be integrated to the running data ingestion pipeline. Additionally, the query processing component of the framework will be further developed to enable features such as *predictive caching* to anticipate the queries that are likely to be issued next, according to user’s interaction behaviour, and *federated querying* by implementing a *linked data fragments* interface, which boosts system scalability by pushing part of the query computation to the client-side application [56,57].

## Figures and Tables

**Table 1:** API specification for the serving layer (default values are shown in underlined text).

HS queries: GET /airquality/{metric_id}/aggregate/{aggregate}/history	
Path parameters	metric_id: (*required*) oone of the air quality metrics available from the Bel-Air setup (no2|pm25|pm10|o3|…). aggregate: (*required*) one of the available aggregate function (AVG|SUM|COUNT).
Query paramenters	q_polygon: (required) Well-Known Text (WKT) representation of the polygon selected by the user, e.g.: “POLYGON ((1.0 0.0, 1.0 1.0, 0.0 0.0, 1.0 0.0))”. source: tile grid (tiles), street blocks (street_blocks) or raw sensor data (raw). time_res: min|hour|day|month. grid_precision: in case multiple continuous views corresponding to multiple values of geohash precision have been computed, via this parameter it is possible to specify the desired precision for the query at hand (default: 6). from: the start of the query interval as a timestamp in milliseconds. to: the end of the query interval (exclusive) as a timestamp in milliseconds. interval: optionally it is possible to use one of five predefined intervals: 5min|1hour|1day|1week|1month.
ST queries: **GET/airquality/{metric_id}/aggregate/{aggregate}/snapshot**	
Path parameters	Same as for the previous endpoint
Query paramenters	bbox: (*required*) comma-separated string of coordinates corresponding to the bounding box over which the snapshot would be taken. source, time_res, grid_precision: same as for the previous endpoint. snap_ts: timestamp in milliseconds corresponding to the instant the snapshot would be taken.

**Table 2:** Versions of the software used in the experimental setup.

Software	Version
Kubectl	0.15.10
Linux Kernel	4.15.0-66-generic
Operating System	Ubuntu 18.04.3 LTS
Container Runtime Version	containerd://1.2.6
PostgreSQL (TimescaleDB + PostGIS + PipelineDB)	11.5 (1.4.2 + 2.5.2 + 1.0.0)
Apache Kafka	2.3.0
NGINX + uWSGI	1.14.2 + 2.0.17.1
Jetty Server	9.4.20.v20190813
Java (OpenJDK)	14-ea
Python	3.7.5

**Table 3:** Composition of the test workload used for the performance evaluation.

Query Type	# Queries
HS (w/temporal predicate)	90
HS (w/o temporal predicate)	52
ST	80
**Total**	**222**

## Abbreviations

The following abbreviations are used in this manuscript:

API	Application programming interface
CSV	Comma-separated values
JSON	Javascript object notation
RDF	Resource description framework
SQL	Structured query language
